# Supplementary material for: The Target-Defining Attributes Can Determine the Effects of Attentional Control Settings in Singleton Search Mode
Source: Behav Sci (Basel). 2025 Jan 20;15(1):97. doi: 10.3390/bs15010097 (PMC11762503; doi:10.3390/bs15010097)
Supplement: Supplementary file 1 [file behavsci-15-00097-s001.zip › behavsci-3371550-supplementary.pdf]

Supplementary Materials

Table 1. the Accuracy (%)（M ± SD）and Reaction times (ms) (M ± SD) of matching level (S+C+, S+C-, S-C+, S-C-) and cue validity (valid, invalid) in four Experiments.

			S+C+	S+C-	S-C+	S-C-
Exp1	ACC(%)	Valid	93.28 ± 11.89	93.43 ± 11.16	92.36 ± 11.98	93.38 ± 12.43
		Invalid	91.60 ± 10.86	92.38 ± 12.61	90.23 ± 13.58	93.40 ± 12.09
	RT(ms)	Valid	607.07 ± 55.14	590.51 ± 54.44	607.98 ± 54.34	589.39 ± 52.33
		Invalid	616.72 ± 51.18	597.53 ± 58.38	617.93 ± 56.77	596.70 ± 57.62
Exp2	ACC(%)	Valid	89.74 ± 6.20	92.66 ± 5.22	90.18 ± 5.96	92.71 ± 6.23
		Invalid	88.84 ± 7.95	92.08 ± 5.86	87.37 ± 8.06	92.97 ± 4.65
	RT(ms)	Valid	670.53 ± 59.30	658.97 ± 51.26	675.68 ± 57.37	655.46 ± 51.63
		Invalid	675.89 ± 61.12	662.05 ± 52.29	684.54 ± 58.43	659.28 ± 53.82
Exp3	ACC(%)	Valid	94.05 ± 5.21	93.55 ± 5.68	93.60 ± 5.59	94.03 ± 5.93
		Invalid	91.15 ± 6.60	93.63 ± 5.15	91.58 ± 6.14	93.43 ± 5.84
	RT(ms)	Valid	593.59 ± 59.04	579.09 ± 56.57	593.43 ± 64.77	580.73 ± 60.63
		Invalid	601.66 ± 61.77	585.98 ± 61.41	605.90 ± 61.12	583.33 ± 56.04
Exp4	ACC(%)	Valid	93.00 ± 8.14	94.28 ± 6.89	94.74 ± 6.37	94.83 ± 7.38
		Invalid	93.17 ± 8.18	93.87 ± 6.71	91.35 ± 8.40	94.96 ± 6.38
	RT(ms)	Valid	606.59 ± 70.76	607.07 ± 80.27	614.36 ± 76.46	602.74 ± 75.98
		Invalid	612.77 ± 66.68	614.99 ± 84.74	627.35 ± 75.20	604.88 ± 73.56
